# Pathology and molecular epidemiology of *Mycobacterium pinnipedii* tuberculosis in native New Zealand marine mammals

**DOI:** 10.1371/journal.pone.0212363

**Published:** 2019-02-12

**Authors:** Wendi D. Roe, Baukje Lenting, Anna Kokosinska, Stuart Hunter, Padraig J. Duignan, Brett Gartrell, Lynn Rogers, Desmond M. Collins, Geoffrey W. de Lisle, Kristene Gedye, Marian Price-Carter

**Affiliations:** 1 School of Veterinary Science, Massey University, Palmerston North, New Zealand; 2 The Marine Mammal Centre, Sausalito, California, United States of America; 3 AgResearch, Palmerston North, New Zealand; Animal Health Centre, CANADA

## Abstract

*Mycobacterium pinnipedii* causes tuberculosis in a number of pinniped species, and transmission to cattle and humans has been reported. The aims of this study were to: characterize the pathology and prevalence of tuberculosis in New Zealand marine mammals; use molecular diagnostic methods to confirm and type the causal agent; and to explore relationships between type and host characteristics. Tuberculosis was diagnosed in 30 pinnipeds and one cetacean. Most affected pinnipeds had involvement of the pulmonary system, supporting inhalation as the most common route of infection, although ingestion was a possible route in the cetacean. PCR for the RD2 gene confirmed *M*. *pinnipedii* as the causal agent in 23/31 (74%) cases (22 using DNA from cultured organisms, and one using DNA from formalin-fixed paraffin-embedded (FFPE) tissue), including the first published report in a cetacean. RD2 PCR results were compared for 22 cases where both cultured organisms and FFPE tissues were available, with successful identification of *M*. *pinnipedii* in 7/22 (31.8%). In cases with moderate to large numbers of acid-fast bacilli, RD2 PCR on FFPE tissue provided a rapid, inexpensive method for confirming *M*. *pinnipedii* infection without the need for culture. VNTR typing distinguished New Zealand *M*. *pinnipedii* isolates from *M*. *pinnipedii* isolated from Australian pinnipeds and from common types of *M*. *bovis* in New Zealand. Most (16/18) *M*. *pinnipedii* isolates from New Zealand sea lions were one of two common VNTR types whereas the cetacean isolate was a type detected previously in New Zealand cattle.

## Introduction

Tuberculosis is a disease characterized by formation of tuberculous granulomas, and caused by acid-fast bacilli belonging to the *Mycobacterium tuberculosis* complex (MTBC) group. It was first described in pinnipeds as far back as 1913 [1], but it was not until 2003 that the causal agent in marine mammals was recognized as a novel member of the complex, now known as *M*. *pinnipedii* [2]. Since then, pinniped tuberculosis has been described in a range of otariid (eared seal) species, both in the wild and in captivity [2]. Although no large-scale studies of *M*. *pinnipedii* prevalence have been published for wild pinnipeds, tuberculosis is believed to be widespread in several species [3, 4]. The potential host range for *M*. *pinnipedii* infection appears to be broad, with infections reported in multiple non-marine mammal species, including humans [5–7].

New Zealand is home to a number of marine mammals, including two endemic endangered species, the New Zealand sea lion (*Phocarctos hookeri*) and Hector’s dolphin (*Cephalorhynchus hectori*). Two cases of tuberculosis have been reported in New Zealand pinnipeds [8, 9] although the causal mycobacterial species were not confirmed, and *M*. *pinnipedii* has been isolated from tuberculous granulomas in the lymph nodes of seven New Zealand cattle [6]. New Zealand fur seals (*Arctocephalus forsteri*), which are widespread throughout New Zealand and Australia and have been diagnosed with *M*. *pinnipedii* infection [2], were thought to be the most likely vectors for the cattle infections [6]. All of the cattle cases were directly or indirectly exposed to seals, either via beach grazing or via access to waterways connecting with the ocean. New Zealand sea lion and fur seal populations are expanding, bringing them increasingly into contact with humans and with domestic species [10, 11]. Accordingly, there is a need to better understand the distribution, prevalence and nature of *M*. *pinnipedii* infection in New Zealand marine mammals, as this has implications for population management and for inter-species disease transmission.

In collaboration with the New Zealand Department of Conservation, our group maintains an extensive archive of tissues collected during necropsy of marine mammals that are either accidentally caught during fishing operations (‘bycaught’) or found dead around the coastline of mainland New Zealand and at Enderby Island in the Subantarctic. While a tentative diagnosis of tuberculosis can be made histologically on the basis of the presence of acid-fast bacilli within characteristic granulomas, the gold standard for etiological diagnosis is microbiological culture followed by molecular confirmation of species [12]. This however requires specialised laboratory facilities since the cultured organism poses a health risk to staff, and is time-consuming and relatively expensive, with positive culture taking up to six weeks. In addition, where fresh or frozen tissue is not available, diagnosis by culture is impossible, hence the ability to confirm *M*.*pinnipedii* infection using formalin-fixed paraffin-embedded (FFPE) tissues would be useful. Using archived tissues and necropsy data, this study aimed to characterize the gross lesions, histological lesions and prevalence of tuberculosis in New Zealand bycaught and stranded marine mammals; to speciate the causal agent; to investigate relationships between isolates by using variable number tandem repeat/direct repeat (VNTR/DR) typing; and to develop a method to confirm *M*. *pinnipedii* infection using FFPE specimens.

## Materials and methods

### Identification of cases and characterization of lesions

Gross post mortem records held at Massey University were reviewed for 1908 marine mammals examined between January 1999 and January 2017. All samples were collected and held under Department of Conservation permit (permit numbers Rnw/HO/2008/03, 35561-MAR and Rnw/22/2003/182). Cases comprised New Zealand sea lions (n = 1304), New Zealand fur seals (n = 236), Hector’s dolphins (n = 216), common dolphins (*Delphinus delphis;* n = 91), dusky dolphins (*Lagenorhynchus obscurus;* n = 32), bottlenose dolphins (*Tursiops truncatus*; n = 14), leopard seals (*Hydrurga leptonyx;* n = 11), southern elephant seals (*Mirounga leonina;* n = 3) and a Subantarctic fur seal (*Arctocephalus tropicalis;* n = 1). Histological slides were retrieved and reviewed for all animals with a tentative gross diagnosis of tuberculosis, as defined by the presence of caseous or granulomatous lesions in lymph nodes, lungs, mediastinum, pleura, liver and/or spleen [2, 3, 13]. Tissue sections were stained routinely with haematoxylin and eosin (H&E) stain and sections containing granulomatous lesions were also stained with Ziehl-Neelsen (ZN) stain for acid-fast bacilli. Where present, the number of acid-fast bacilli present was graded as ‘few’ (less than 1 per 10–20 100x fields), ‘moderate’ (acid-fast bacilli present individually or in small clusters in several 40x fields) or ‘large numbers’ (numerous large clusters of acid-fast bacilli present and easily visible at 20x magnification).

### Culture and initial molecular analysis

Where available, frozen tissues were retrieved and prepared for mycobacterial culture as follows. Tissues were homogenized using Ten Broek grinders, decontaminated with NaOH, and inoculated into Bactec 12B (Becton Dickinson, Franklin Lakes, New Jersey, USA) or the Bactec MGIT 960 automated broth-based system (Becton Dickinson, Cockeysville, Maryland 21030, USA). Solid media culture was on Middlebrook 7H11 (7H11P) medium which was supplemented with pyruvate and prepared as described previously [14]. All media were incubated at 37°C. Bactec 12B vials and liquid 7H9 were incubated for a maximum of 30 days, MGITs for 42 days and solid media for 90 days. Microbial growth in liquid culture was detected by the release of CO_2_, (Bactec) or by fluorometric detection of O_2_ consumption (MGIT) Positive Bactec 12B and MGIT cultures were checked for contamination by inoculating blood agar plates and for mycobacteria by the microscopic examination of Ziehl Neelsen-stained smears.

Preliminary identification of mycobacterial isolates was based on use of a DNA probe specific for the *M*. *tuberculosis* complex (Accuprobe, Gen-Probe, San Diego, California, USA). Mycobacterial isolates were confirmed as *M*. *pinnipedii* by PCR using *M*. *pinnipedii*–specific multiplex for the determination of the absence (168-base pair [bp] product, *M*. *pinnipedii*) or presence (293-bp product) of the DNA region of difference (RD2) as described by Warren *et al*. [15]. For analysis of FFPE tissues, DNA was extracted from 10 μm thick sections using the High Pure FFPET DNA isolation kit (Roche, Basal, Switzerland) as per the manufacturer’s instructions, and the RD2 assay conducted as above.

### Molecular typing of isolates

DNA extraction and typing using a combination of nine VNTR assays and the direct repeat (DR) locus, as described by Price-Carter et al. [16], was carried out on cultured organisms from all confirmed marine mammal *M*. *pinnipedii* cases. [6] (6) (6) (6) *M*. *pinnipedii* isolates from the marine mammals in this study were compared with a reference database of *M*. *bovis* and *M*. *pinnipedii* isolates that had been subjected to the same assays. Since results at the DR2 locus were not reliable, they were excluded from the dendrogram analysis. Each isolate was defined by a string of 10 integers (the VNTR/DR1 profile), corresponding to the number of repeats found at the 10 different VNTR/DR loci (MIRU40, ETRD, ETRC, ETRE, NZ2, QUB18, QUB11a, QUB26, DR2, and QUB3232) used in the analysis. Isolate W12/0358-6 (Case 12) was excluded from the dendrogram analysis because it contained a mixture of 2 different sized QUB3232 PCR products. The *M*. *pinnipedii* VNTR profiles were analyzed using the MIRU-VNTR*plus* web based application [17, 18]. Clustering analysis used a distance matrix generated using the most conservative coefficient (a categorical distance) and a dendrogram was constructed with the unweighted pair-group method with arithmetic mean (UPGMA). The categorical distance is the number of markers with a different allele (number of repeats) divided by the total number of VNTR’s used. Missing data was ignored for the distance calculations.

## Results

### General findings

Forty-three of 1908 (2%) marine mammals were identified as suspect cases of tuberculosis. Tuberculosis was confirmed in 31/1908 (1.6%) marine mammals (30 pinnipeds and 1 cetacean) (Table 1), based on the presence of caseous or granulomatous lesions in conjunction with intralesional acid-fast bacilli and/or a positive result for *Mycobacterium* sp. using the MGIT system. Six of the suspect cases (all New Zealand sea lions) were unable to be investigated further due to lack of archived tissues. Six further suspect cases (3 New Zealand fur seals and 3 New Zealand sea lions) were negative on both acid fast stains and culture. In 9 of the 12 non-confirmed cases, only a single lymph node was affected. The remaining 3 had granulomatous lesions in the liver (n = 1), lungs and thoracic lymph nodes (n = 1) or superficial lymph nodes and pleura (n = 1).

**Table 1:** Case details and gross lesions present in marine mammals diagnosed with tuberculosis.

				Tuberculosis lesions						
Case No.	Species	Proven-ance	Sex/ Age class	Super-ficial LNs Head/ Neck	Lung parench-yma	Intra-thoracic LNs	Pleural effusion	Pericar-ditis	Abdominal organ granulomas	Cause of death
1	NZSL	NZ/FD	MA	+	+^m^	+	+	-	LN, Li, Sp, Ki, Int	TB
2	NZSL	NZ/FD	MA	+	+	+	+	-	LN, Li, Sp	TB
3	NZSL	NZ/FD	MA	+	-	+	-	-	Li	TB
4	NZSL	NZ/FD	MA	+	+^m^	+	+	+	LN, Li, Ki, Int	Neoplasia
5	NZSL	NZ/FD	MA	-	+	+	+	-	LN	TB
6	NZSL	NZ/FD	MA	+	+	+	+	-	-	TB
7	NZSL	NZ/FD	MA	-	+	+	-	-	Li	TB
8	NZSL	NZ/FD	MA	-	+	+	-	-	-	TB
9	NZSL	NZ/FD	MA	+	+	+	-	-	LN	TB
10	NZSL	End/FD	FA	+	+^m^	+	+	-	LN, Ov, Ut, P	TB
11	NZSL	End/FD	MA	-	-	+	+	-	-	Predation
12	NZSL	End/FD	FA	+	+	+	-	-	-	TB
13	NZSL	End/FD	FA	+	+	+	-	-	LN	TB
14	NZSL	End/FD	FA	+	+	+	+	-	LN	TB
15	NZSL	End/FD	FA	-	+	+	-	-	-	Trauma
16	NZSL	End/FD	MS	-	-	+	-	-	-	Enteritis
17	NZSL	End/FD	MS	-	n/a	n/a	n/a	n/a	LN, Li	TB
18	NZSL	End/FD	FA	-	+	+	+	+	-	TB
19	NZSL	End/FD	FA	-	+	+	+	+	-	TB
20	NZSL	End/FD	FA	-	-	+	+	-	-	TB
21	NZSL	End/FD	FA	-	-	-	+	+	LN, Sp	TB
22	NZSL	End/FD	FS	-	+	+	+	-	-	TB
23	NZSL	End/FD	MA	+	-	+	+	-	LN	TB
24	NZSL	Auck/B	MA	-	+	+	-	-	-	Bycatch
25	NZSL	Auck/B	FA	+	-	-	-	-	-	Bycatch
26	NZSL	Auck/B	MA	-	-	+	-	-	-	Bycatch
27	NZSL	Auck/B	FA	-	+	+	+	+	-	Bycatch
28	NZFS	NZ/B	MA	+	+	+	+	-	-	Bycatch
29	NZFS	NZ/FD	FA	+	+	+	-	-	-	TB
31	NZFS	NZ/FD	MJ	+	+	+	+	-	LN, Li, Sp	TB
30	HD	NZ/FD	FA	+	+	+	-	-	LN	TB

NZ = New Zealand; SL = sea lion; FS = fur seal; HD = Hector’s dolphin; FD = found dead; B = bycatch; End = Enderby Island; Auck = Auckland Island shelf; MA = male adult; FA = female adult; MS = male subadult; FS = female subadult; MJ = male juvenile; LNs = lymph nodes; Li = liver; Sp = spleen; Ki = kidney; Int = intestines; Ov = ovaries; Ut = uterus; P = peritoneum; + = lesion present;— = lesion absent; +^m^ = miliary lung lesions; n/a = not able to be assessed due to scavenging; TB = tuberculosis

Of the confirmed cases, all affected pinnipeds were either New Zealand sea lions (n = 27) or New Zealand fur seals (n = 3), while the cetacean was a Hector’s dolphin. Tuberculosis was the diagnosed cause of death in 22/31 (71%) cases, and was a concurrent finding in the remaining nine (29%) cases. The majority of affected animals were either adults (n = 25) or sub-adults (n = 5), with a single case in a juvenile.

New Zealand sea lions were the most common species represented in the study, making up 1304/1908 (68%) marine mammals in the database, and comprising 27/31(87%) confirmed cases of tuberculosis. Further demographic details of this group are shown in Table 2.

**Table 2:** Provenance and age group details for New Zealand sea lions.

Location	Classification	Pup/juvenile	Subadult/ adult
Auckland Island region	Bycatch	0/0 (0%)	4/198 (2%)
New Zealand mainland	Found dead	0/13 (0%)	9/32 (28%)
Enderby Island	Found dead	0/986 (0%)	14/75 (19%)

Demographic and age group details for New Zealand sea lions included in the study, showing number confirmed to have tuberculosis (numerator) and total examined (denominator).

### Gross and histological lesions

**Fig 1:**
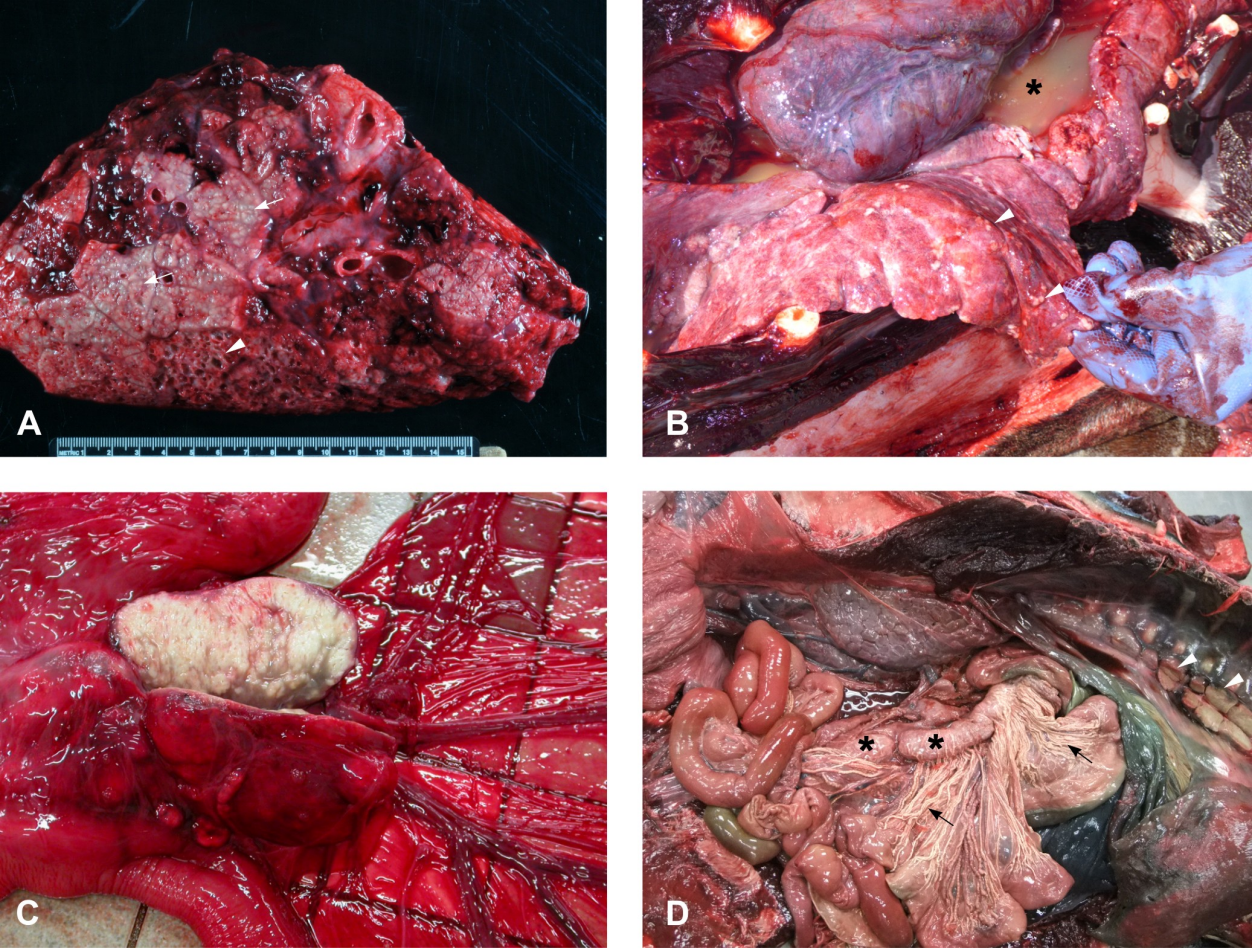
Gross pathology of *M*. *pinnipedii* infection. **A.** Cut surface of lung from a severely affected New Zealand sea lion. There are areas of lung consolidation containing multifocal to coalescing caseating granulomas (arrows). In some areas these have central areas of liquefaction and tissue loss (arrowhead). **B.** Miliary lesions: there are numerous small (2-4mm diameter) granulomatous lesions (arrowheads) scattered throughout the lung fields of this New Zealand sea lion, along with watery turbid fluid within the thoracic cavity (pleural effusion; asterisk). **C**. Cut surface of an enlarged, pale, firm, nodular mesenteric lymph node from a New Zealand sea lion with tuberculosis. **D.** Tuberculosis in a Hector’s dolphin. The mesenteric (asterisk) and intra-thoracic (arrowheads) lymph nodes are enlarged and pale and the mesenteric lymphatics are pale and thickened (arrows).

Details of gross lesions are included in Table 1. Pulmonary granulomas were present in 22/31 cases (71%). In the most severely affected animals, up to 80% of the pulmonary parenchyma was replaced by multifocal to coalescing areas of caseous necrosis, frequently with foci of liquefaction. Large areas of consolidation were common, and airways often contained mucoid to purulent exudate (Fig 1A). Three cases had miliary lesions throughout the pulmonary parenchyma (Fig 1B). In six of the nine cases without pulmonary parenchyma involvement, granulomas were present in other thoracic organs (lymph nodes, mediastinum, pleura and/or pericardium). In one case (Case 17) the thoracic organs could not be assessed as they had been removed by scavenging birds. Seventeen animals had pleural effusions (Fig 1B), characterized by up to 2 litres of turbid grey or blood-tinged effusion, occasionally containing clots of fibrin. The mediastinum in these cases was thickened and proliferative, frequently with villous to nodular projections, and the pleura was similarly thickened.

Affected lymph nodes were either enlarged, soft and diffusely pale (Fig 1C and 1D) or contained multifocal to coalescing opaque cream foci, sometimes with areas of liquefaction. Three animals (Cases 3, 23 and 31) had caseous granulomas in one neck lymph node with adjacent soft tissue abscessation and a draining sinus tract. Two otherwise healthy bycaught New Zealand sea lions (Cases 25 and 26) had involvement of a single lymph node (one axillary node and one bronchial node).

**Fig 2:**
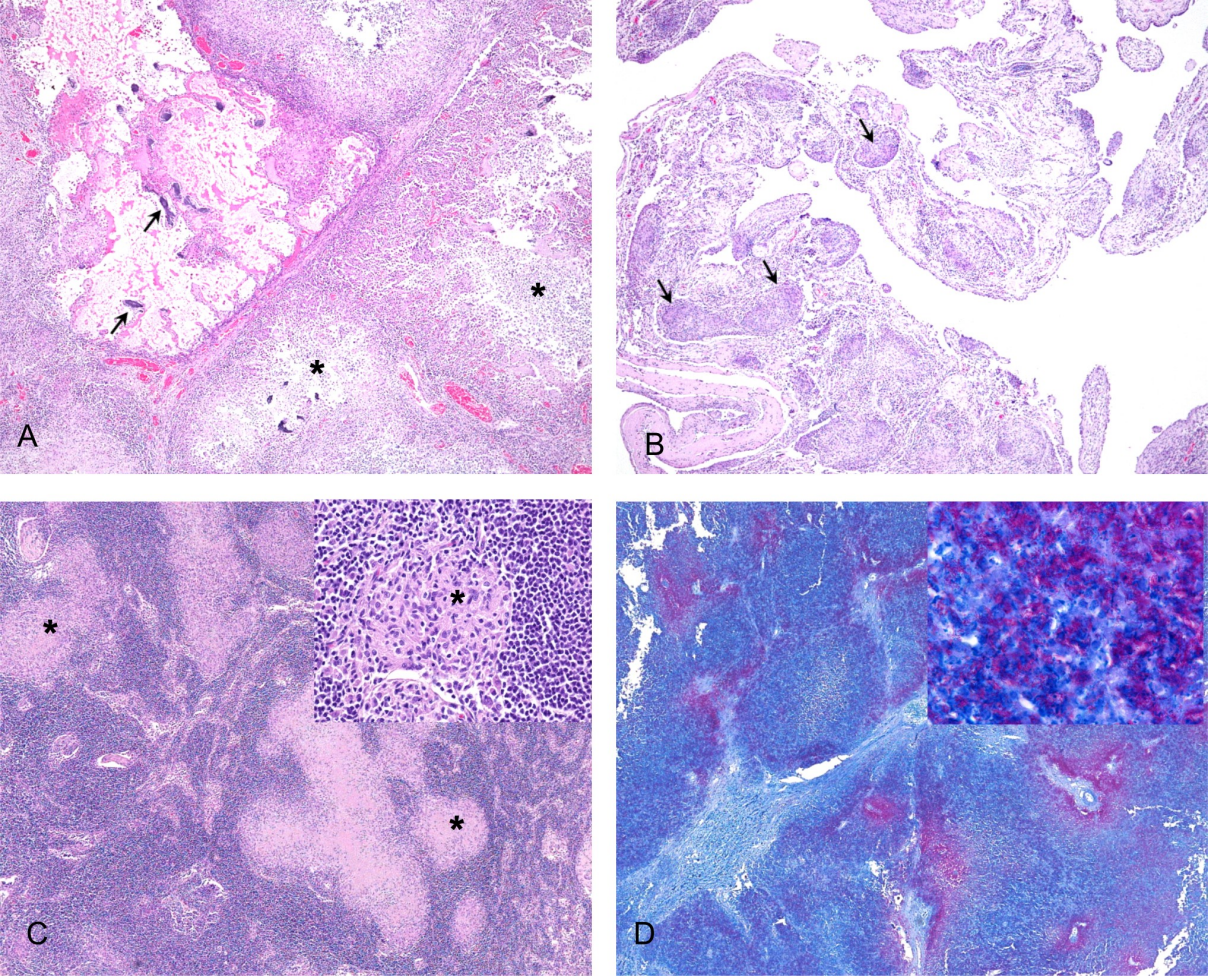
Histopathological lesions present in marine mammals diagnosed with tuberculosis due to *Mycobacterium pinnipedii*. **A.** Lung of a sea lion showing focally extensive destruction of normal parenchyma (top left corner) with mineralisation of alveolar septae. Adjacent tissue shows multifocal granulomas with central areas of necrosis (asterisks), 4x, H&E. **B.** Mediastinal tissue from a sea lion, which is thickened by diffuse granulomatous inflammation and multiple small granulomas (arrows), 4x, H&E. **C.** Lymph node from a New Zealand sea lion, showing multifocal to coalescing granulomas (asterisks), 4x. Inset shows a single small granuloma (asterisk) surrounded by normal lymphoid tissue, 40x. Both stained with H&E. **D.** Superficial lymph node from a Hector’s dolphin. Areas of necrosis are outlined by large numbers of red-stained acid-fast bacilli, 4x. Inset shows high power magnifaction of acid-fast bacilli, 100x. Both stained with Ziehl-Neelsen stain.

Histological features of tuberculous lesions are shown in Fig 2. The lungs in affected pinnipeds had multifocal to coalescing granulomas that obliterated normal pulmonary architecture (Fig 2A). Small granulomas (less than 2-3mm diameter) were characterised by aggregates of epithelioid macrophages admixed with low to moderate numbers of lymphocytes, plasma cells, and plump fibroblasts in a loose fibrous stroma. Larger granulomas contained central zones of necrosis surrounded by macrophages with fewer lymphocytes and plasma cells, and variable amounts of fibrous tissue either within the granulomatous inflammation or surrounding individual granulomas. Many of these larger granulomas had liquefied necrotic centres, which were frequently accompanied by destruction of bronchiolar walls and presence of exudate within airways. Mineralization was infrequent, and where present was predominantly within alveolar septae. Multinucleated giant cells were rare. The pleural surface of the lungs was thickened by granulomatous (n = 17) to pyogranulomatous (n = 1 (case 5)) inflammation, fibrin, necrosis and fibrosis in 18/31 (58%) animals. Similar changes were present on the pericardium (n = 5) and the mediastinum (n = 17) (Fig 2B), with formation of villonodular projections. Tuberculous lesions contained few to many fine filamentous acid-fast bacilli, predominantly within or surrounding necrotic areas.

Lesions in affected lymph nodes (Fig 2C) and abdominal organs were similar to the pulmonary granulomas described above, with most granulomas larger than 2–3 mm in diameter having central areas of necrosis. Mineralization of these lesions was rare.

The single cetacean diagnosed with tuberculosis (case 30) presented with enlargement of multiple superficial, intra-thoracic and intra-abdominal lymph nodes, extremely prominent ‘corded’ intestinal lymphatics (Fig 1D), and scattered small (1–3 mm) granulomas in the pulmonary parenchyma. Extremely large numbers of acid-fast bacilli were present within granulomas (Fig 2D).

### Molecular analysis

Mycobacterial culture followed by DNA extraction and amplification of RD2 confirmed *M*. *pinnipedii* as the causal agent of tuberculosis in 22/31 (71%) cases. This agent was also confirmed in one further case, for which cultured organisms were not available for analysis, by amplification of RD2 from FFPE tissues. Overall therefore, *M*. *pinnipedii* was confirmed as the causal tuberculosis agent in 23/31 (74%) cases. For the remaining eight animals with tuberculosis, the causal agent could not be confirmed due to negative culture (n = 4) or unavailability of tissues (n = 4).

PCR for RD2 using DNA extracted from lesional FFPE tissues was positive in 8/31 (25.8%) cases in total (see Table 3). These included 7 of 22 (31.8%) cases in which *M*. *pinnipedii* had been confirmed by RD2 PCR from culture, and the case described above, where an MTBC was cultured but the isolate was not available for RD2 assay. FFPE tissues were more likely to be positive where there were moderate to large numbers of acid-fast bacilli (6/8 with moderate to large numbers vs 2/23 with few or none; P = 0.001; Fisher’s exact test). The FFPE assay was negative on all culture-negative cases.

**Table 3:** RD2 results using DNA from formalin-fixed tissues and cultures.

Case no.	AFB	RD2 from FFPE	RD2 from culture
30	+++	positive	positive
23	+++	positive	positive
1	+++	positive	positive
3	+++	positive	positive
7	+++	negative	not done^a^
9	+++	positive	not done^b^
10	+++	negative	not done^c^
17	+++	negative	Positive
5	++	positive	Positive
2	+	positive	Positive
4	+	negative	Positive
12	+	negative	Positive
15	+	negative	Positive
16	+	negative	not done^c^
18	+	negative	not done^c^
29	+	negative	Positive
6	+	negative	not done^a^
8	+	negative	not done^a^
13	+	negative	Positive
19	+	negative	Positive
20	+	negative	Positive
21	+	negative	Positive
22	+	negative	Positive
24	+	negative	Positive
26	+	positive	Positive
27	+	negative	not done^a^
28	+	negative	Positive
31	+	negative	Positive
25	+	negative	not done^c^
11	none	negative	positive
14	none	negative	positive

Comparison of RD2 PCR assay results for FFPE tissues and cultured organisms, showing the relative number of acid-fast bacilli present on lesional FFPE tissues. For cases where RD2 assays were not conducted on cultured organisms, the reason is indicated by a superscript.

AFB = acid-fast bacilli; FFPE = formalin-fixed paraffin embedded tissue

^a^ = no tissue available

^b^ = culture positive but no DNA archived

^c^ = culture negative; + = few AFB (<1/10-20 100X fields); ++ = moderate; AFB present individually or as small clusters in several 40X fields; +++ = large clusters of AFB easily visible at 20X

Although a valuable marker for distinguishing between *M*. *bovis* isolates [16], our DR2 PCR assay primers are not robust for amplification of this locus in marine mammal samples. Observed products were often very faint and not repeatedly detected, suggesting that the primer binding site in *M*. *pinnipedii* may be different to that in *M*. *bovis*. DR2 results were therefore excluded from the dendrogram analysis. All other loci were reliably detected in all analyzed *M*. *pinnipedii* isolates and closely resembled previously characterised isolates from New Zealand cattle [6].

**Fig 3:**
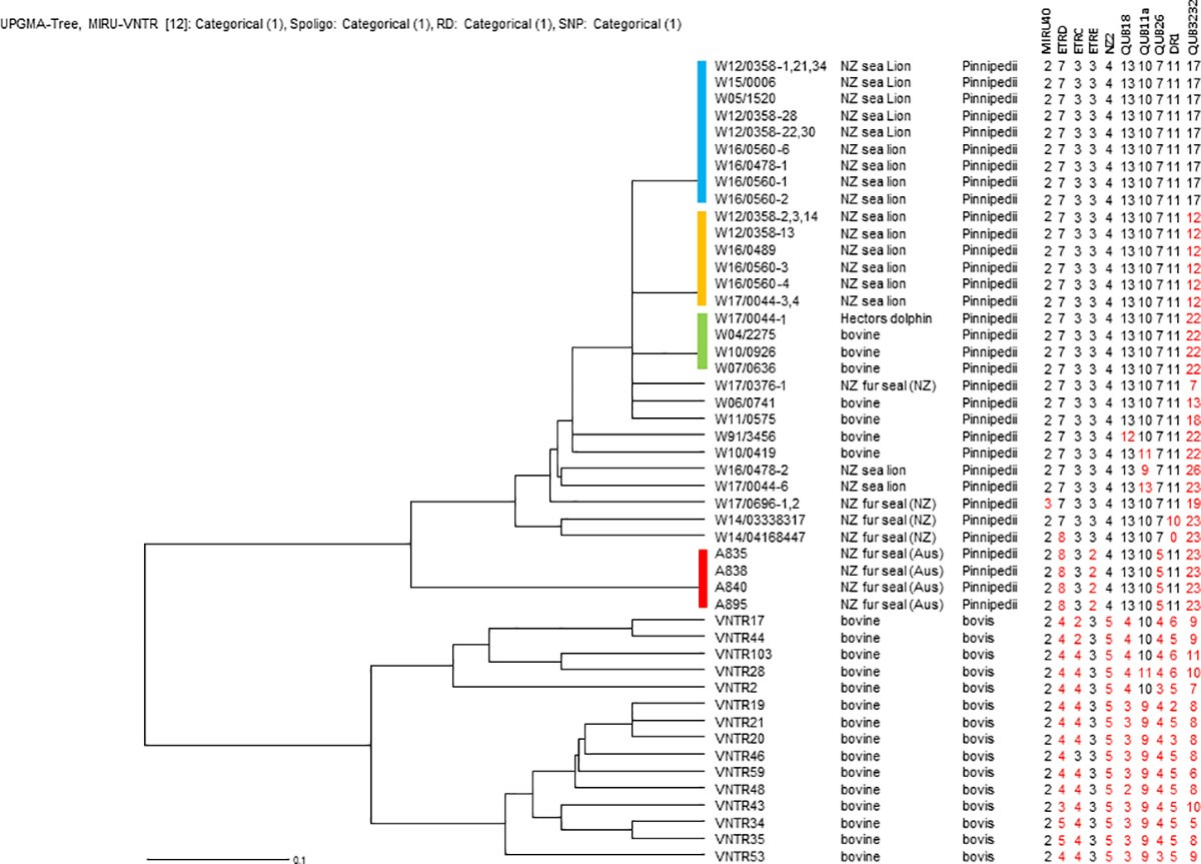
Dendrogram. Nine variable number tandem repeat and two direct repeat loci were used to compare *Mycobacterium pinnipedii* strains isolated from cattle and pinnipeds in New Zealand with selected *M*. *pinnipedii* isolates from Australian pinnipeds and selected *M*. *bovis* isolates from New Zealand cattle.

VNTR/DR typing was carried out on 21 of the marine mammal isolates from this study, and compared with *M*. *pinnipedii* isolates from our database: eight from New Zealand cattle and four from Australian pinnipeds (all previously published cases in New Zealand fur seals resident in Australia [2]. The dendrogram in Fig 3 illustrates the relationship of New Zealand marine mammal isolates to other New Zealand *M*. *pinnipedii* and *M*. *bovis* isolates at the nine VNTR loci and at the DR1 locus. All of the New Zealand marine mammal isolates had the same sized ETRC (three repeats rather than the two or four copies observed in most New Zealand *M*. *bovis* isolates), NZ2 (four repeats rather than five in common New Zealand *M*. *bovis*) and Q26 (seven repeats rather than three or four), that was typical of the previously characterised New Zealand bovine *M*. *pinnipedii* isolates and distinguished these pinniped isolates from common New Zealand *M*. *bovis* types. All but one also had the larger DR1 locus (10 or 11 repeats rather than 6 or less) that was typical of New Zealand *M*. *pinnipedii*. It was not possible to amplify this locus from one of the pinniped isolates, possibly because the primer binding site had changed or the DR1 locus was lost in this isolate. Like the New Zealand *M*. *pinnipedii* isolates, the Australian *M*. *pinnipedii* isolates were distinct from common New Zealand *M*. *bovis* types since they had four rather than five copies of NZ2 and 13 copies of QUB18. The Australian *M*. *pinnipedii* isolates differed from the New Zealand *M*. *pinnipedii* isolates since they had two rather than three repeats at the ETRE locus, and five rather seven repeats at the QUB26 locus.

Three distinct *M*. *pinnipedii* VNTR/DR types, which differed from one another only at the QUB3232 locus, were isolated from more than one animal. Most New Zealand sea lions (16/18) were one of two common types. Nine animals (all New Zealand sea lions) had the most common VNTR/DR type with 17 copies of QUB3232, while six New Zealand sea lions had the next most common type with 12 copies of QUB3232. Although none of the types detected in fur seal isolates were the same as those detected in New Zealand cattle, three of the previously characterised bovine isolates and the cetacean isolate had the next most common VNTR/DR type with 22 copies of QUB3232; none of the pinnipeds in this study had this type. Four isolates differed from these three main VNTR/DR types only at the QUB3232 locus (two bovine, one fur seal (Case 28) and one sea lion (Case 12). The four identified New Zealand fur seal types were distinct by one or more VNTR loci from all other types. Within the less variable loci, eight (rather than seven) copies of ETRD were present only in Australian pinnipeds and one New Zealand fur seal. As discussed below, finding eight copies of ETRD in both Australian and New Zealand pinnipeds is likely to be an example of homoplasy since the New Zealand isolates with this size PCR product were more similar to New Zealand isolates when compared at other loci.

## Discussion

The results of this study confirm the presence of *M*. *pinnipedii* infection in New Zealand fur seals, New Zealand sea lions, and a Hector’s dolphin. Although the overall prevalence of marine mammal tuberculosis in this study is low (31/1908 cases; 1.6%), this disease appears to be a common cause of mortality in sub-adult and adult New Zealand sea lions, causing 8/32 (25.0%) mainland New Zealand and 11/75 (14.6%) Enderby Island sea lion deaths. Assessing the true prevalence of tuberculosis infection in free-living marine mammals is problematic. Bycaught pinnipeds can theoretically be considered to represent a healthy adult population, since they are accidentally caught during their normal foraging activities. Tuberculosis was diagnosed in 4/198 (2.0%) bycaught New Zealand sea lions and 1/136 (0.7%) bycaught New Zealand fur seals in this study; however this may be an underestimate of the true prevalence of infection, since small lesions in a single lymph node are likely to be missed during post mortem examination. Jurczynski et al. [19] found that multiple testing modalities (serology, computed tomography and examination of sputum) were necessary to reliably detect *M*. *pinnipedii* infection in live captive species, but this approach is impractical for free-living pinnipeds. Commercially available serological tests have been used for ante mortem diagnosis of tuberculosis in a number of wildlife species [12, 19]; however to date none of these tests have been validated for use in New Zealand marine mammals.

The predominance of pulmonary involvement in the pinniped cases presented here indicates that inhalation is the primary route of infection in New Zealand fur seals and sea lions, as has been reported for other pinniped species (reviewed by Kriz *et al*., 2011[3]). In humans most infections occur through direct aspiration of bacteria expectorated in the respiratory secretions of an actively infected individual [20]. If the same pathway occurs in pinnipeds, transmission is likely to be enhanced by close contact, for example congregation and harem formation during the breeding season or at shared haul-out sites at other times of year. The route of infection for the Hector’s dolphin in this study is less clear. In this case the widespread involvement of lymph nodes throughout the body indicates extensive lymphatic spread, but the site of entry is uncertain. Involvement of the intestinal lymph nodes and lymphatics suggests the possibility of ingestion as a route of infection. Although environmental survival of *M*. *pinnipedii* has not been investigated, survival of other MTBC species has been demonstrated in environmental substrates (water, saline, soil and sediment) [21, 22], as well as invertebrates [23] and amoebae [24]. To the knowledge of the authors, *M*. *pinnipedii* infection has only been diagnosed in one other cetacean, a bottlenose dolphin *(Tursiops truncatus*) from Australia (Cousins D, unpublished data) but the lesion distribution was not reported. Intriguingly, the VNTR type of the dolphin isolate in this current study was the same as that previously detected in New Zealand cattle, and was distinct from that found in New Zealand sea lions. The prevalence and route of mycobacterial infection in cetaceans warrant further investigation.

The gross and histological lesions found in this study are similar to those described for other pinniped species [2, 3, 13, 25], and are characterized by granulomas that frequently show central caseous necrosis, with variable amounts of fibrosis. Mineralisation and multinucleated giant cells are infrequent, while pleural effusion and granulomatous inflammation of serosal surfaces within the thorax are common features. The presence of histological pulmonary lesions with liquefaction of granulomas and destruction of bronchiolar walls accompanied by the presence of exudate within airways is likely to be associated with bacterial shedding from airway secretions, and further supports respiratory secretions as a major pathway of transmission in pinnipeds. The presence of granulomas within the intestinal mucosa (n = 2) and abdominal lymph nodes (n = 11) of pinnipeds in this study raises the possibility that fecal shedding of mycobacteria may also occur in some cases.

This study found that FFPE tissues can, in some circumstances, be used to confirm *M*. *pinnipedii* infection using a PCR assay for RD2. This method is likely to be most useful as a rapid, specific, inexpensive test for diagnosis of *M*. *pinnipedii* infection in cases with moderate to large numbers of acid-fast bacilli on histological examination. Several false negatives were apparent in the current study, particularly in paucibacillary cases, however a larger sample size would be required to more accurately determine the sensitivity of this test. While culture of MTBC organisms is slow and requires special handling and isolation facilities due to the risk of zoonotic infection, it remains the gold standard for *M*. *pinnipedii* diagnosis, and is essential if subsequent genotyping is required using currently established methods.

Molecular methods such as VNTR analysis provide a rapid and a relatively simple means of comparing isolates from different MTBC species, host species and geographical regions [26]. The ability to discriminate depends on the VNTR/DR loci employed for the analysis and the extent to which these loci vary in different bacterial isolates. Previous analyses of MTB complex bacteria by VNTR have shown that some loci change relatively frequently and others infrequently or not at all [26]. A combination of slow and rapidly changing loci has proven optimal for distinguishing New Zealand *M*. *bovis* types [16]. The scheme used here involves 10 loci (nine VNTR and one DR) that were published previously for bovine isolates of *M*. *pinnipedii* [6]. In the current study, three distinct VNTR/DR types were detected in isolates from more than one animal. These types differed from one another only at the QUB3232 locus which is known to be the most rapidly-changing of these 10 loci [6], but has proved to be a useful epidemiological tool for distinguishing New Zealand *M*. *bovis* isolates. The finding that isolates from 18 of the 22 (82%) VNTR/DR-typed New Zealand marine mammals are identical at all but the most rapidly changing locus most likely reflects the fact that these isolates descended from a common ancestor, as well as the slow rate of change of most of these markers in mycobacteria. As suggested by Ahlstrom *et al*. [27], when isolates are compared by both VNTR and whole genome sequencing (WGS) analysis some of the detected similarities in type may be due to genetic homoplasy (the occurrence of the same copy number in epidemiologically distinct isolates). Homoplasy in our isolates will be most evident by WGS, therefore a selection of New Zealand *M*. *pinnipedii* isolates are currently being characterised by WGS in order to better define the relationship suggested by VNTR/DR analysis. In addition, this will allow us to develop assays based on single nucleotide polymorphisms (SNPs) that can be used to type isolates from FFPE extracts.

## Conclusions

This study shows that tuberculosis due to *M*. *pinnipedii* is a comparatively common cause of death in subadult and adult New Zealand sea lions, is present in New Zealand fur seals, and is a potential risk for Hector’s dolphins. PCR assay for the RD2 locus using DNA extracted from FFPE tissues provides a rapid, safe, and inexpensive means of confirming *M*. *pinnipedii* infection in cases where moderate to large numbers of acid-fast bacilli are present in lesions. Although VNTR/DR typing distinguished New Zealand *M*. *pinnipedii* isolates from Australian *M*. *pinnipedii* and from common New Zealand *M*. *bovis* types, there was a high degree of similarity between isolates from the majority of affected marine mammals. The ability to study epidemiology and transmission pathways of this disease would be enhanced by the development of WGS methods, which would facilitate the use of SNP assays to more accurately distinguish important clusters. In addition, this will enable further investigation of the relationship between the cetacean isolate reported here and those previously found in New Zealand cattle.

## Supporting information

S1 Table Details for marine mammals diagnosed with tuberculosis. (XLSX)
